# Activation of Protein Serine/Threonine Phosphatase PP2Cα Efficiently Prevents Liver Fibrosis

**DOI:** 10.1371/journal.pone.0014230

**Published:** 2010-12-06

**Authors:** Lirui Wang, Xu Wang, Jing Chen, Zhengyi Yang, Liang Yu, Lihong Hu, Xu Shen

**Affiliations:** 1 State Key Laboratory of Drug Research, Shanghai Institute of Materia Medica, Chinese Academy of Sciences, Shanghai, China; 2 E-Institutes of Shanghai Municipal Education Commission, Shanghai Jiaotong University School of Medicine, Shanghai, China; The University of Hong Kong, Hong Kong

## Abstract

**Background:**

Over-activation of TGFβ signaling pathway and uncontrolled cell proliferation of hepatic stellate cells (HSCs) play pivotal roles in liver fibrogenesis, while the protein serine/threonine phosphatase PP2Cα was reported to negatively regulate TGFβ signaling pathway and cell cycle. Our study aimed to investigate the role of PP2Cα in liver fibrogenesis.

**Methodology/Principal Findings:**

The effects of PP2Cα activation on liver fibrosis were investigated in human HSCs and primary rat HSCs *in vitro* using western blotting, real-time PCR, nuclear translocation, cell viability and cell cycle analyses. The antifibrogenic effects in carbon tetrachloride (CCl_4_)- and bile duct ligation (BDL)-induced mice *in vivo* were assessed using biochemical, histological and immunohistochemical analyses. The results demonstrated that activation of PP2Cα by overexpression or the new discovered small molecular activator NPLC0393 terminated TGFβ-Smad3 and TGFβ-p38 signaling pathways, induced cell cycle arrest in HSCs and decreased α-smooth muscle actin (α-SMA) expression, collagen deposition and hepatic hydroxyproline (HYP) level in CCl_4_- and BDL-induced mice.

**Conclusions/Significance:**

Our findings suggested that PP2Cα activation might be an attractive new strategy for treating liver fibrosis while the small molecular activator NPLC0393 might represent a lead compound for antifibrogenic drug development. Moreover, our study might provide the first evidence for the role of PP2C family members in the fibrotic disease.

## Introduction

Liver fibrosis is a major public health threat causing portal hypertension, liver failure, and risk of hepatocellular carcinoma. Hepatic stellate cells (HSCs) play critical roles in liver fibrogenesis. Once intoxicated by stimuli, quiescent HSCs could transdifferentiate into activated HSCs which secrete some proinflammatory and profibrogenic cytokines such as tumor necrosis factor alpha (TNFα) and transforming growth factor beta (TGFβ), leading to over-accumulation of extracellular matrix (ECM) and altered matrix degradation. Meanwhile, these cytokines further activate HSCs and enhance their proliferation and survival, thus exacerbating fibrogenesis [1]. Recently, emerging strategies against liver fibrosis have been proposed, such as selective antagonization of CB1 cannabinoid receptor [2], targeting 5-hydroxytryptamine (5-HT) class of receptors [3], inhibition of Toll-like receptor 4 (TLR4) [4], and activation of STAT1 [5], *etc*. However, the efficient strategies are still lacking due to the complicated pathogenesis associated with this disease [6].

Protein serine/threonine phosphatases (PS/TPs) dephosphorylate phosphoserine/phosphothreonine-containing proteins and comprise three structurally distinct families: phosphoprotein phosphatases (PPPs), metal-dependent protein phosphatases (PPMs), and the aspartate-based phosphatases represented by FCP/SCP (TFIIF-associating component of RNA polymerase II CTD phosphatase/small CTD phosphatase). Protein phosphatase 2C, which belongs to PPM family, is a structurally and functionally distinct group of enzymes that currently contain about 22 different family members. The members of this family are distinguished by their monomeric property and dependency on Mg^2+^ and Mn^2+^ [7]. It should be noted that except the oncoprotein PP2Cδ (also known as Wip1) [8], [9], all the other members from this family have been identified as tumor suppressors based on their inhibition of cell growth and cellular stress signaling [10], [11].

Protein phosphatase 2C alpha (PP2Cα; EC 3.1.3.16), the most extensively characterized member of PP2C family, plays an important role in TGFβ, cell growth, stress and inflammation signaling [10], [12]–[15]. PP2Cα was reported to dephosphorylate Smad2/Smad3 to block TGFβ signaling pathway [12], activate p53 and dephosphorylate Cdk2/Cdk6 to induce cell cycle arrest [13], [14], inhibit p38 and JNK signaling pathways to prevent stress [16] and dephosphorylate IKappa B kinase β (IKKβ) to prevent inflammatory response [15]. Recently, the potential role of PP2Cα in tumorigenesis has been revealed [11], whereas its function in the fibrotic disease still remains unknown. The current study therefore aimed to investigate the role of PP2Cα in liver fibrosis by assessing the effects on TGFβ signaling pathway and cell cycle of HSCs and ECM expression in mouse models. Our findings suggest that PP2Cα activation might be a promising new strategy for the treatment of liver fibrosis.

## Results

### Activation of PP2Cα inhibited TGFβ-Smad3 and TGFβ-p38 signaling pathways in HSCs

**Figure 1:**
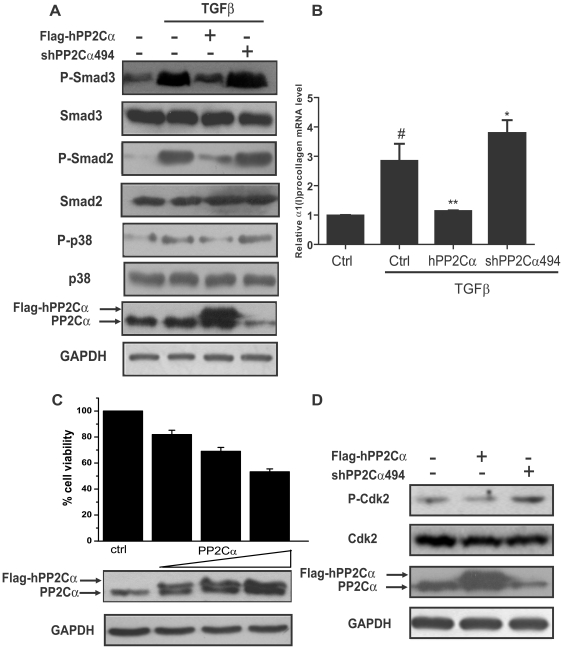
Activation of PP2Cα inhibited both TGFβ-Smad3 and TGFβ-p38 signaling pathways and induced cell cycle arrest in HSCs. (A) Flag-hPP2Cα, shPP2Cα494 and control vectors were electransfected into LX-2 cells. At 48 h post-transfection, 2 ng/ml of TGFβ was used to stimulate the cells for 1 h. Cells were harvested and proteins were immunoblotted with the indicated antibodies. (B) At 24 h post-transfection with hPP2Cα and shPP2Cα494, LX-2 cells were stimulated with TGFβ (2ng/ml) for another 24 h. Cells were harvested for real-time PCR experiment. ^#^P<0.001 compared with non-TGFβ-treated group; *P<0.05, **P<0.01 compared with TGFβ-treated group tranfected with control vector. (C) Cell viability was assessed by MTT assay at 48 h post-transfection with increasing concentrations of hPP2Cα (upper panel). Increasing expressions of PP2Cα were verified by western blotting (lower panel). (D) Cells were harvested at 48 h post-transfection with hPP2Cα and shPP2Cα494 and total cell extracts were analyzed by western blotting.

Since Smad3 was regarded as the main mediator of TGFβ-induced fibrotic response [12], [17], we first assessed the impact of PP2Cα on TGFβ-induced Smad3 phosphorylation in human hepatic stellate cell line LX-2 cells. As shown in Figure 1A, TGFβ stimulated Smad3 phosphorylation, while the stimulation was obviously decreased after PP2Cα overexpression and slightly enhanced with PP2Cα knock-down by shPP2Cα494. Similarly, the TGFβ-induced Smad2 phosphorylation was reduced with PP2Cα overexpression and mildly increased with PP2Cα knock-down. Considering that p38 was also reported to mediate TGFβ-induced fibrotic effects [16], [18], we examined the effect of PP2Cα on TGFβ-induced p38 phosphorylation. The result revealed that TGFβ stimulated the phosphorylation of p38 and this stimulation could be regulated by PP2Cα overexpression or knock-down (Figure 1A).

We next studied whether PP2Cα affected TGFβ-induced collagen transcription that was reported to be up-regulated by Smad3 and p38 phosphorylations [17], [18]. Consistently, the results indicated that TGFβ increased α1(I) procollagen mRNA transcription, whereas PP2Cα overexpression aborted the stimulatory effect of TGFβ while PP2Cα knock down enhanced it (Figure 1B). These findings demonstrated that overexpression of PP2Cα suppressed TGFβ-Smad3 and TGFβ-p38 signaling pathways in HSCs.

### Activation of PP2Cα induced cell cycle arrest in HSCs

The regulation of PP2Cα on cell cycle in several cell lines was reported previously [11], [13], [14]. Consistent with these reports, our work demonstrated that overexpression of PP2Cα inhibited LX-2 cell viability in a dose-dependent manner (Figure 1C).

Cdk2, an important regulatory protein of G1-S transition, was reported to mediate PP2Cα induced cell cycle arrest [13]. Therefore, to verify whether the cell viability loss was due to cell cycle arrest induced by PP2Cα, we examined Cdk2 phosphorylation. The result (Figure 1D) indicated that overexpression of PP2Cα attenuated Cdk2 phosphorylation, while knock down of PP2Cα enhanced it. These results suggested that PP2Cα induced cell cycle arrest in LX-2 cells through down-regulating Cdk2 phosphorylation.

### Identification of NPLC0393 as a small molecular PP2Cα activator

**Figure 2:**
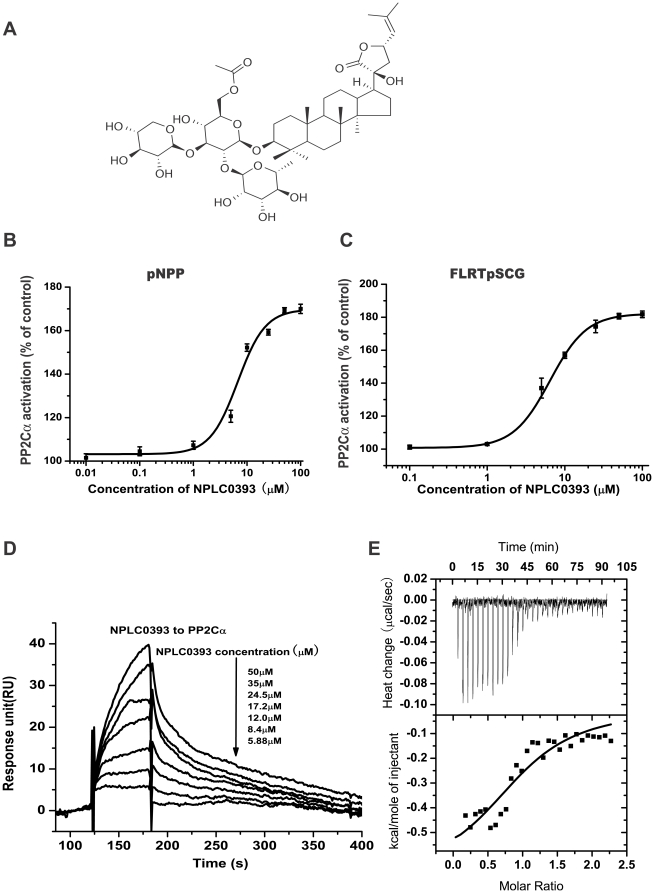
Identification of NPLC0393 as a small molecular PP2Cα activator. (A) Chemical structure of NPLC0393. (B, C) NPLC0393 activated the recombinant human PP2Cα activity using pNPP (B) and phosphopeptide FLRTpSCG (C) as substrates. Data are expressed as the mean ± S.D. of three independent experiments. (D) Binding affinity of NPLC0393 to PP2Cα as evaluated by Biacore 3000. Sensorgrams obtained from NPLC0393 injection over the immobilized PP2Cα surface. NPLC0393 was injected for 60s, and dissociation was monitored for more than 120s. (E) ITC analysis of NPLC0393/PP2Cα interaction.

To further verify the therapeutic potential of PP2Cα, we identified a small molecular PP2Cα activator, NPLC0393, through a reconstituted *in vitro* PP2Cα phosphatase assay (Figure 2A) [19]. The result revealed that NPLC0393 dose-dependently increased PP2Cα activity with an EC_50_ value of 6.72 µM using pNPP as substrate (Figure 2B). Additionally, we further confirmed the enhancement of PP2Cα activity by NPLC0393 using the phosphopeptide substrate FLRTpSCG, which is derived from AMP-activated protein kinase and was previously reported to be a good substrate for PP2Cα [14]. The result indicated that NPLC0393 also increased PP2Cα activity in a dose-dependent manner, with an EC_50_ value of 6.43 µM (Figure 2C).

Subsequently, we confirmed the direct binding of NPLC0393 to PP2Cα through surface plasmon resonance (SPR) technology based assay. The dissociation equilibrium constant (K_D_) was thus determined as 19.2 µM (Figure 2D). In addition, the isothermal titration calorimetry (ITC) was also applied to analyze the stoichiometry and thermodynamics of NPLC0393/PP2Cα interaction by titrating NPLC0393 to PP2Cα (Figure 2E). The results revealed that the stoichiometric ratio was 1.05±0.03, implying that a single molecule of NPLC0393 could interact with one molecule of PP2Cα. Furthermore, the determined K_D_ was approximately 14.7 µM, similar to the SPR result. Notably, the change in Gibbs' free energy (ΔG) resulting from NPLC0393/PP2Cα interaction was driven primarily by a favorable entropy (TΔS, 5.93 kcal/mol), compared with the enthalpy (ΔH, −0.669 kcal/mol), suggesting that NPLC0393/PP2Cα binding was mainly mediated by the increase of the buried surface area rather than the polar interactions (Figure 2E).

To assess the targeting specificity of NPLC0393, we evaluated the effects of NPLC0393 on two representative mammalian Ser/Thr phosphatases (PP1 and PP2A) and one typical Tyrosine phosphatase (PTP1B). The results in Table 1 thereby indicated that NPLC0393 had no obvious activities against these three tested phosphatases, further suggesting its good specificity against PP2Cα.

**Table 1:** Selectivity of NPLC0393 against a panel of phosphatases *in vitro*.

Phosphatases	activity (% of control)
PP2Cα	170
PP1	99
PP2A	92
PTP1B	105

Collectively, our results demonstrated that NPLC0393 as a specific small molecular activator of PP2Cα might be used as a potential probe to elucidate the biological significance of PP2Cα in relevant diseases.

### NPLC0393 inhibited TGFβ-Smad3 and TGFβ-p38 signaling pathways in HSCs

**Figure 3:**
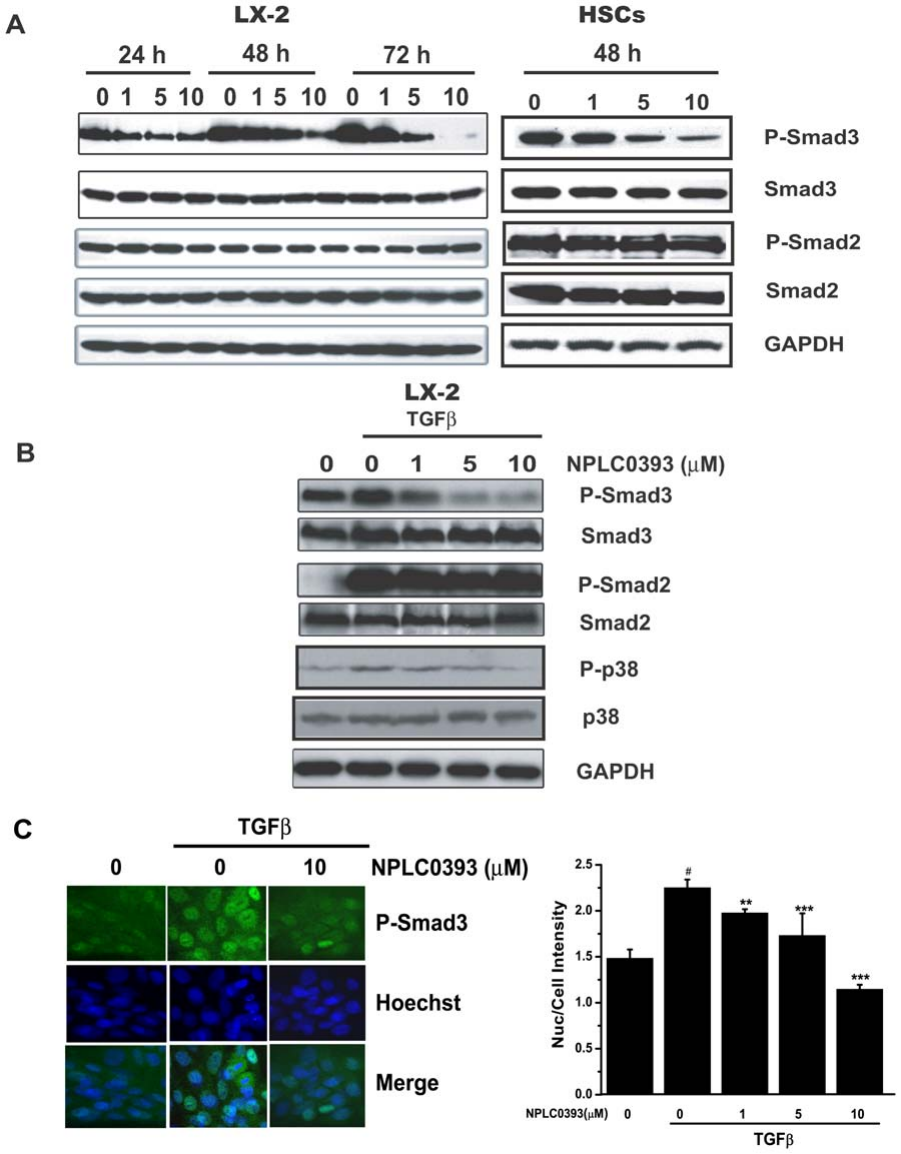
NPLC0393 reduced TGFβ-Smad3 and TGFβ-p38 phosphorylations in HSCs. (A,B) LX-2 cells and the isolated primary rat HSCs were treated with increasing concentrations of NPLC0393 for indicated time points. Cells were harvested and the total cell extracts were analyzed by western blotting. (C) LX-2 cells were treated with NPLC0393 for 48 h followed by TGFβ (1ng/ml) stimulation for another 1 h. Effects of NPLC0393 on the nuclear translocation of P-Smad3 were assessed by Immunofluorescence experiment. Images were taken by IN Cell Analyzer 1000 and quantified by counting six random chosen fields in each well. Each treatment was performed in three wells. ^#^P<0.001 compared with non-TGFβ-treated group; **P<0.01; ***P<0.001 compared with TGFβ-treated group with vehicle treatment.

**Figure 4:**
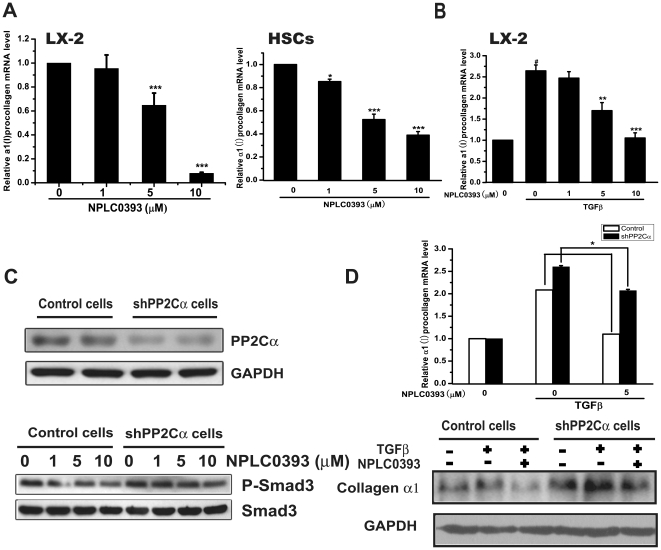
NPLC0393 decreased TGFβ-induced α1(I) collagen expression in HSCs. (A) LX-2 cells and the isolated primary rat HSCs were treated with increasing concentrations of NPLC0393 for 48 h. Cells were harvested for real-time PCR experiment. ***P<0.001 compared with vehicle group. (B) LX-2 cells were treated with NPLC0393 and TGFβ for 48 h, cells were then harvested and the total RNA was extracted. ^#^P<0.001 compared with non-TGFβ-treated group; **P<0.01; ***P<0.001 compared with TGFβ-treated group with vehicle treatment. (C) Characterization of stable LX-2 cell line expressing shPP2Cα by western blot analysis (upper panel). Control and shPP2Cα cells were treated with increasing concentrations of NPLC0393 for 24 h and harvested for Western blotting (lower panel). (D) Control and shPP2Cα cells were treated with NPLC0393 and TGFβ for 48 h and harvested for western and real-time PCR analysis. Significant difference of the reduction on α1(I) procollagen mRNA by NPLC0393 in shPP2Cα cells versus that in control cells, *P<0.05.

The effects of NPLC0393 on TGFβ-Smads and TGFβ-p38 signaling pathways were assessed in LX-2 cells and primary rat hepatic stellate cells (HSCs). The results indicated that NPLC0393 decreased Smad3 phosphorylation in both time- and dose-dependent manners (Figure 3A), and the TGFβ-induced Smad3 and p38 phosphorylations were also reduced by NPLC0393 treatment (Figure 3B). Moreover, NPLC0393 inhibited Smad3 nuclear localization (Figure 3C), which was reported to depend on its phosphorylation [12]. Additionally, it should be pointed out that NPLC0393 rendered no evident influence on basal or TGFβ-induced Smad2 phosphorylation (Figure 3A,B). Finally, NPLC0393 decreased basal and TGFβ-induced α1(I) procollagen mRNA expression (Figure 4A,B). Furthermore, NPLC0393 failed to exert the above effects in PP2Cα stable knock-down cells (shPP2Cα cells) (Figure 4C,D), thus confirming that these effects of NPLC0393 were mediated by PP2Cα. Altogether, these findings indicated that treatment of NPLC0393 could block TGFβ-Smad3 and TGFβ-p38 signaling pathways through inhibiting Smad3 and p38 phosphorylations and Smad3 nuclear localization.

### NPLC0393 induced cell cycle arrest in HSCs

**Figure 5:**
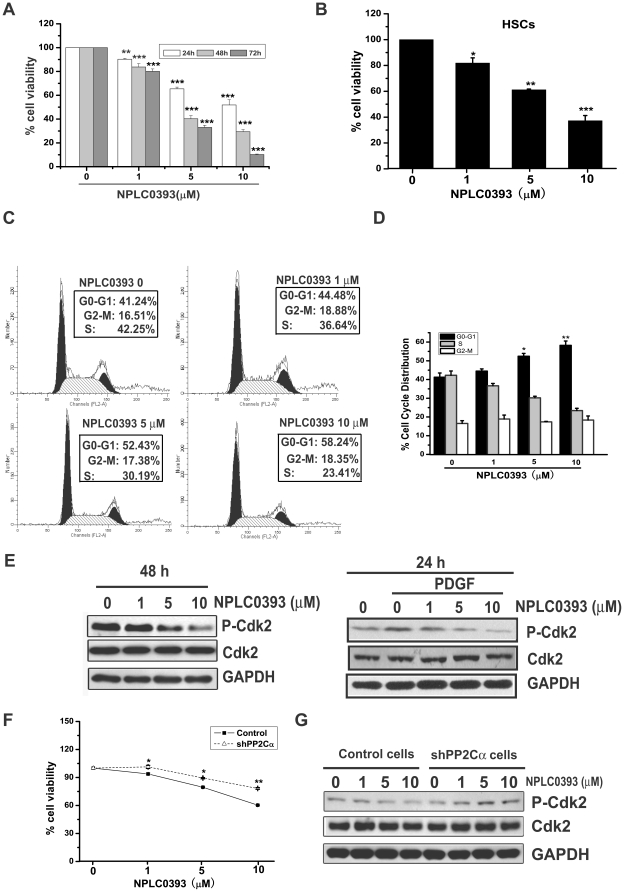
NPLC0393 induced cell cycle arrest in HSCs. (A,B) LX-2 cells and the isolated primary rat HSCs were treated with increasing concentrations of NPLC0393 for the indicated time points. MTT assay was performed to assess the effects of NPLC0393 on cell viability. The values were indicated as relative units normalized to the control. *P<0.05; **P<0.01; ***P ≤ 0.001 compared with control group at the indicated time point. (C, D) LX-2 cells were exposed to increasing concentrations of NPLC0393 for 48 h. Then cells were harvested and the cell-cycle distribution was analyzed by Flow cytometry analysis. (E) LX-2 cells were treated as described in Figure 3C. Effect of NPLC0393 on Cdk2 phosphorylation was assessed by western blotting. For the PDGF-induced Cdk2 phosphorylation, LX-2 cells were cultured to confluence and growth-arrested for 24 h in DMEM with 10% FBS, and then for an additional 24 h treatment with NPLC0393 and PDGF (10 ng/ml) in DMEM plus with 0.2% FBS. (F) Control and shPP2Cα cells were treated with increasing concentrations of NPLC0393 for 24 h. Effects of NPLC0393 on cell viability in shPP2Cα cells and control cells were assessed by MTT assay. Significant difference of the reduction on cell viability by NPLC0393 in shPP2Cα cells versus that in control cells at indicated dose, *P<0.05, **P<0.01. (G) Cells were treated as described in Figure 3F and harvested for Western blotting.

The impact of NPLC0393 on cell cycle was also examined in LX-2 and primary rat HSCs cells. As indicated in Figure 5A and B, NPLC0393 decreased HSCs cell viability in a time- and dose-dependent manner as assessed by MTT assay. To figure out whether such cell viability reduction was due to cell cycle arrest, we next carried out flow cytometry analysis. The results demonstrated that 48h incubation of NPLC0393 dose-dependently induced G1 phase arrest in LX-2 cells (Figure 5C,D). Analysis of cell cycle regulatory proteins revealed that NPLC0393 decreased phosphorylation of Cdk2 in LX-2 cells (Figure 5E, left). Considering that Platelet-Derived Growth Factor (PDGF) could stimulate cell proliferation by increasing Cdk2 phosphorylation [20], we also examined the effect of NPLC0393 on PDGF-induced p-Cdk2 level in LX-2 cells. The result displayed that NPLC0393 obviously inhibited PDGF-induced Cdk2 phosphorylation in a dose-dependent manner (Figure 5E, right). Moreover, the effects of NPLC0393 on cell cycle were subsequently studied in shPP2Cα cells. The results showed that NPLC0393 failed to decrease cell viability (Figure 5F) and Cdk2 phosphorylation (Figure 5G) in shPP2Cα cells compared with control cells. Therefore, these findings indicated that activation of PP2Cα by NPLC0393 induced cell cycle arrest in HSCs.

### NPLC0393 attenuated liver fibrogenesis *in vivo*

**Figure 6:**
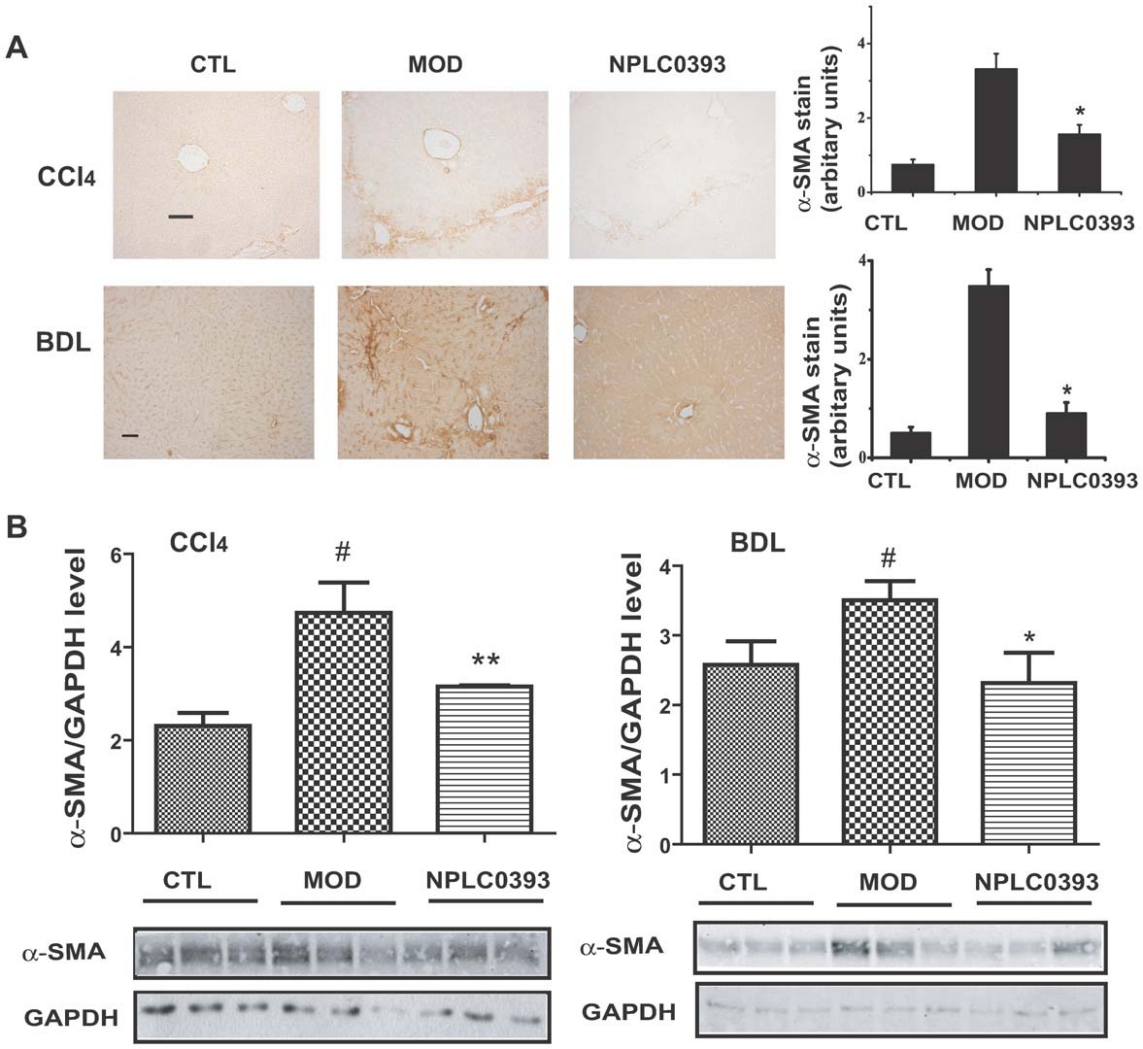
NPLC0393 attenuated CCl_4_- and BDL-induced α-SMA expressions *in vivo*. (A) Expression of α-SMA in CCl_4_- and BDL-intoxicated mice was evaluated by immunohistochemical staining, and quantified by counting five random chosen high-power fields. Scale bar, 50 µm. n = 9 for control (CTL), model (MOD) and 2.5 mg/kg NPLC0393-treated (NPLC0393) mice. (B) α-SMA expression was also assessed by western blotting and quantified from three independent experiments, *P<0.05, **P<0.01. n = 3 for control (CTL), model (MOD) and 2.5mg/kg NPLC0393-treated (NPLC0393) mice.

**Figure 7:**
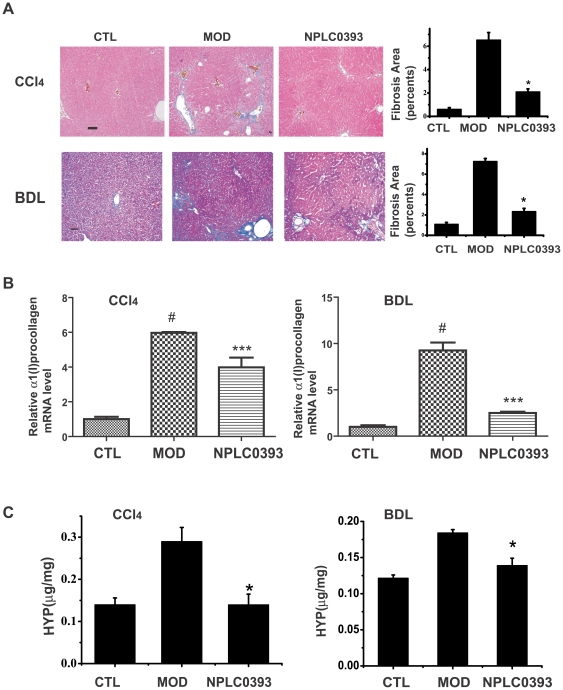
NPLC0393 attenuated CCl_4_- and BDL-induced collagen expressions *in vivo*. (A) Collagen deposition in livers was evaluated by Masson staining and determined by image quantification. Scale bar, 100 µm. (B) Collagen mRNA expression was examined by real-time PCR analysis. ***P ≤ 0.001, n = 3 for control (CTL), model (MOD) and 2.5 mg/kg NPLC0393-treated (NPLC0393) mice. (C) Hydroxyproline content in liver was also measured. Significant difference versus model group, *P<0.05. n = 9 for control (CTL), model (MOD) and 2.5 mg/kg NPLC0393-treated (NPLC0393) mice.

To further investigate the anti-liver fibrosis potential of PP2Cα, two different mouse models bearing liver fibrosis were treated with the PP2Cα activator NPLC0393. Compared with the vehicle group, treatment of NPLC0393 (2.5 mg/kg) rendered no obvious influence on the serum alanine transaminase (ALT), aspartate transaminase (AST) levels or the liver histology, implying that NPLC0393 was little toxic *in vivo* (data not shown). As shown in Figure 6A and B, 2.5 mg/kg of NPLC0393 administration decreased α-SMA expression in both CCl_4_ and BDL-induced liver fibrosis mice. In addition, Masson staining of collagen indicated that NPLC0393 reduced the fibrosis area in both models (Figure 7A). The CCl_4_ and BDL-induced α1(I) procollagen mRNA levels were also decreased in the NPLC0393-treated mice (Figure 7B). Moreover, NPLC0393 administration declined the ECM marker, hydroxyproline (HYP) content in the two kinds of liver fibrosis mice (Figure 7C). It should be also noted that NPLC0393 decreased the ALT and AST levels in both CCl_4_ and BDL-induced liver fibrosis mice, suggestive of its protective function in liver injury (data not shown). Taken together, all these results thus suggested that NPLC0393 as a PP2Cα activator could significantly attenuate liver fibrogenesis in both CCl_4_- and BDL-induced liver fibrosis mice.

## Discussion

In recent years, PP2C family has received an extensive research interest for its wide implications in the critical signaling pathways associated with human diseases [8], [10]–[16], [21], [22]. PP2Cα, a representative member of PP2C family, was determined to possess tumor-suppressing properties [11]. However, its potential role in fibrotic disease still remains untouched. Considering that liver fibrogenesis is always accompanied with TGFβ over-activation, stress, HSCs excessive proliferation and severe inflammatory response [1], [6], we thus assume that PP2Cα might be also connected with liver fibrogenesis for its negative role in TGFβ, stress, cell cycle and inflammatory signaling pathways [12]–[16]. Here, we demonstrated that PP2Cα activation could terminate TGFβ signaling pathway and simultaneously induce cell cycle arrest in HSCs, leading to significant anti-fibrogenic effects both *in vitro* and *in vivo*, although we could not exclude the possibility that the anti-fibrotic effects of PP2Cα activation might be also mediated by reduction of stress and inflammatory response, which is however beyond our current study.

The crucial role of TGFβ signaling in liver fibrogenesis has been widely recognized [1], [23]. Several anti-TGFβ signaling pathway-targeted strategies were recently proved effective, such as inhibition of latent TGFβ activation or prevention of TGFβ binding to its receptor [24]. These strategies, however, mainly involved large molecular inhibitors (e.g. monoclonal antibodies and antisense oligonucleotides) against TGFβ receptor which might block the systemic immunosuppressive effects of TGFβ [24], [25]. The current anti-fibrogenic reports concerning small molecular inhibitors of TGFβ signaling are only restricted to the inhibitors of TGFβ type I receptor kinase [26]–[28]. In our work, we determined that the natural product NPLC0393 as a specific small molecular PP2Cα activator could efficiently alleviate liver fibrosis. Therefore, our work is expected to provide new insights into the understanding of TGFβ signaling inhibition-based anti-liver fibrogenesis research, while the discovered small molecular PP2Cα activator NPLC0393 might be used as a potential lead compound for anti-liver fibrotic drug discovery.

Interestingly, although Smad2 and Smad3 were both shown to be dephosphorylated by PP2Cα [12], our study revealed that NPLC0393 only selectively dephosphorylated Smad3 without altering Smad2 phosphorylation. Based on the different roles of Smad3 and Smad2 in TGFβ signaling [29], [30] and the fact that Smad3, but not Smad2, mediates the liver fibrosis response [17], we thereby propose that NPLC0393 might supply a promising interest in the treatment of liver fibrosis with high specificity, although the detailed mechanism of such specificity needs to be further investigated. Additionally, consistent with the previous report [16], we uncovered that PP2Cα overexpression or NPLC0393 treatment not only decreased the TGFβ-induced Smad3 phosphorylation but also reduced the TGFβ-induced p38 phosphorylation. Therefore, we assume that the decreased α1(I) collagen expression induced by PP2Cα and NPLC0393 might result from the inhibition of both TGFβ-Smad3 and TGFβ-p38 signaling pathways. Although TGFβ1 transcription was reported to be Smad3-dependent [17], the undetectable decrease of TGFβ1 mRNA expression in NPLC0393 treated liver fibrosis mice might be due to the other signaling pathways besides TGFβ-Smad3, which are also involved in TGFβ1 expression.

As indicated, apart from blocking TGFβ signaling, reducing HSCs was also proved effective in preventing liver fibrogenesis [31], [32]. Here, we determined that PP2Cα activation induced cell cycle arrest of HSCs through decreasing P-Cdk2, thus leading to the evident antifibrotic effects as evaluated in CCl_4_- and BDL-induced mouse models. By considering the well characterized anti-proliferative effects of PP2C family members [22], we thus suggested that our findings might gain insights into their potential roles in the treatment of fibrotic diseases that are always associated with excessive proliferation of activated stellate cells.

To confirm the function of PP2Cα activation on liver fibrogenesis in mice, we carried out two mice models. One is toxic fibrosis model induced by CCl_4_ and the other is biliary fibrosis model induced by BDL. These two models are mediated by different mechanisms. The CCl_4_-induced liver fibrosis begins with inflammatory response which activates HSCs leading to the eventual accumulation of ECM, while the production of ECM in the BDL-induced model is not from inflammatory response which is not so evident in these mice [33], [34]. Notably, our current study has revealed that activation of PP2Cα reduced α-SMA expression, collagen deposition and HYP level in both models, further suggesting that PP2Cα activation exhibited efficient antifibrogenic effects.

To date, quite few compounds targeting PP2Cα have ever been reported although the relevant catalytic mechanism and crystal structure regarding this phosphatase have been elucidated [19], [35], [36]. Considering the potent biological functions of PP2Cα, we randomly screened our in-house natural product library (∼10,000 compounds) against the recombinant human PP2Cα phosphatase for identifying small molecular PP2Cα regulators (inhibitor or activator). The natural product NPLC0393 was thus determined as a specific PP2Cα activator. It should be also pointed out that the mRNA and protein levels of PP2Cα in HSCs were not affected by NPLC0393 (data not shown), further suggesting that NPLC0393 implemented its antifibrotic effects through enhancing PP2Cα enzymatic activity. NPLC0393 is a triterpene saponin extracted from *Gynostemma pentaphyllum*, which is widely used in the treatment of liver disease [37]–[40]. Our findings are thus expected to bring new insights into the potential pharmacological mechanism for this popular traditional herbal medicine, while NPLC0393 might represent a lead compound for antifibrogenic drug development.

Conclusively, our work has indicated that PP2Cα activation not only terminated TGFβ-Smad3 and TGFβ-p38 signaling pathways but also inhibited cell proliferation in hepatic stellate cells. The fact that PP2Cα activation by NPLC0393 remarkably prevented liver fibrogenesis in CCl_4_- and BDL-induced mice, has further confirmed that PP2Cα activation could be a promising strategy for treating liver fibrosis.

## Materials and Methods

### Ethics statement

All the animal related procedures were performed according to the ethical guidelines of Animal care and use committee, Shanghai Institute of Materia Medica, Chinese Academy of Sciences. Permit numbers: SCXK (HU) 2007-0005; SYXK (HU) 2008-0049. This study was approved by Science and Technology Commission of Shanghai Municipality.

### Animals

C57/BL6 male mice at 8-week age were obtained from Shanghai SLAC Laboratory Animal Co. Ltd. The CCl_4_-induced liver fibrosis was generated by intraperitoneal injection of CCl_4_ (0.5 ml/kg, diluted 1∶10 in olive oil) twice weekly, alternating with an isovolumetric dose of 5% ethanol diluted in PBS 5 times per week [2]. NPLC0393 was dissolved in Tween-80 and intraperitoneal injected daily. Groups were as follows (n = 9): mice given olive oil and NPLC0393 (control); mice given CCl_4_, ethanol and Tween-80 (model); mice given CCl_4_, ethanol and treated with 2.5 mg/kg of NPLC0393 (NPLC0393). After 4 weeks, animals were starved overnight and executed 48 h after the last CCl_4_ injection.

The BDL-induced liver fibrosis was constructed by transecting the common bile duct between two ligations after midline laparotomy as described [2]. Groups were as follows (n = 9): mice receiving sham operation and Tween-80 (control); mice receiving BDL and Tween-80 (model); mice receiving BDL and treated with NPLC0393 (NPLC0393). Mice were sacrificed after 2 weeks. Liver samples were either fixed in buffered formalin or snap frozen in liquid nitrogen and stored at −80°C until use.

### Histological and immunohistochemical analysis

Livers were fixed in 4% paraformaldehyde, embedded in paraffin and sectioned. Immunohistochemical staining of α-SMA was performed to quantify activated HSCs. Masson staining for collagen was used to quantify fibrosis area. The results were analyzed by Image-Pro Plus software (MediaCybernetics, France). Images of five fields were taken for each section with 9 mice in each group.

### Hepatic hydroxyproline determination

Hepatic hydroxyproline content was measured using hydroxyproline detection kit (Jiancheng Institute of Biotechnology, Nanjing, China) according to the manufacturer's instruction. The results (µg/mg liver) were calculated according to the standard curve of hydroxyproline.

### Primary HSCs isolation, cell lines, culture and treatment

Primary HSCs were isolated from normal rat liver (male Sprague–Dawley rats, 400–450 g) as described [41]. Cells were cultured in Dulbecco's minimum essential medium (DMEM; GIBCO/Invitrogen) containing 10% fetal bovine serum (FBS; GIBCO/Invitrogen). All the experiments were performed using 6-day culture-activated HSCs whose activation was verified by α-SMA expression using western blotting. Human hepatic stellate cell line LX-2 [42] and HEK293T Phoenix-ampho retrovirus packaging cells (ATCC) were cultured in DMEM supplemented with 10% FBS in 5% CO2 at 37°C. TGFβ and PDGF were from Sigma.

### Cell transfection

Human PP2Cα and shPP2Cα494 (for PP2Cα expression knock down assay) were electransfected into LX-2 cells as described [43] using Amaxa® Cell Line Nucleofector® Kit T (Lonza).

### Establishment of stable LX-2 cell line expressing shPP2Cα

pSRG vector and pSRG-shPP2Cα494 construct were transfected into 293T Phoenix-ampho retrovirus packaging cells. After 48 h, viral supernatant was collected, filtered, and supplemented with polybrene (8 µg/ml). LX-2 cells were infected with viral supernatant. At 48h post-infection, infected cells were selected with puromycin (3 µg/ml). After selection for 5 days, cells were collected and verified by western blotting [12].

### Cell viability assay

Cell viability was evaluated using MTT (Sigma) assay as previously described [44].

### Cell cycle analysis

Cell cycle was analyzed as previously described [44]. The samples were assayed with a FACS Calibur instrument and the data were analyzed with CellQuest 3.1 Software.

### Nuclear translocation

Nuclear translocation was assessed by immunofluorescence experiment as described [25]. The images were taken by IN Cell Analyzer 1000 and the data were analyzed with Nuclear Translocation analysis module [45].

### Western blotting

Primary antibodies used were phospho-Smad3 (Ser423/425), Smad3, phospho-Smad2 (Ser465/467), Smad2, phospho-p38 (Thr180/Tyr182), p38, phospho-Cdk2 (Thr160) and Cdk2 (Cell Signaling Technology), PP2Cα (Abcam), α-SMA (BOSTER, China), Collagen α1 Type I (Santa cruz), GAPDH (KangChen, China). Western blotting was performed according to the manufacturers' instructions.

### Real-time PCR

Extraction of total RNA and synthesis of complementary cDNA were performed as described [46]. Real-time PCR was performed using SYBR Premix Ex Taq (TaKaRa) on DNA Engine Opticon TM2 System (MJ Research, Waltham, MA, USA). The primer pairs for human *β-actin*, human *α1(I) procollagen*, rat *18S* were designed as described [2], [42]. The primer pairs for rat *procollagen α1(I):* 5′CACTCAGCCCTCTGTGCC3′ (sense) and 5′ ACCTTCGCTTCCATACTCG 3′ (antisense). The primer pairs for mouse *procollagen α1(I):* 5′ACGGCTGCACGAGTCACAC3′ (sense) and 5′GGCAGGCGGGAGGTCTT3′ (antisense).

The PCR cycle was 95°C for 5 seconds, 58°C for 20 seconds and 72°C for 20 seconds.

### Identification of human PP2Cα activation by NPLC0393

NPLC0393 was isolated and purified as previously described [47]. Human PP2Cα was expressed in *E. coli* with C-terminal 6-His tag, and batch-purified using Ni-NTA resin according to the manufacturer's instruction (Qiagen). The assay was carried out in a reaction buffer containing 50 mM Tris-HCl, pH 7.0, 10 mM MnCl_2_ [19]. NPLC0393 was dissolved in DMSO as a stock solution and diluted in reaction buffer to the final concentration. PP2Cα was diluted in reaction buffer as appropriate to 10 µg/ml, and reactions were started by the addition of 4 mM pNPP (Sigma), incubated with varying concentrations of NPLC0393 for 2 h at room temperature, and stopped with a solution containing 1 N NaOH. The effect of NPLC0393 on PP2Cα dephosphorylation of pNPP was determined by monitoring the absorbance change recorded at 410 nm, with 1% DMSO as a control.

With phosphopeptide FLRTpSCG (HD Biosciences; China) as the substrate [14], the reaction buffer containing 50 mM Tris-HCl, pH 7.0, 30 mM MgCl_2_ was used. PP2Cα was diluted in reaction buffer as appropriate to 10 µg/ml and incubated with varying concentrations of NPLC0393 for 2 h at room temperature. Then the reaction was started with 500 µM FLRTpSCG for 30 min and terminated by adding 100 µl of malachite green/ammonium molybdate reagent (upstate). Color development was allowed to proceed for 15 minutes at room temperature. Measurements were taken at 630 nm using microplate spectrophotometer (Bio-Rad). The effect of NPLC0393 on PP2Cα dephosphorylation of FLRTpSCG was determined by monitoring the absorbance change recorded at 630 nm, with 1% DMSO as control.

For selectivity assay, PP1 and PP2A were bought from Upstate. PTP1B was purified using Ni-NTA resin according to the manufacturer's instruction (Qiagen). The effects of NPLC0393 on these phosphatases dephosphorylation of pNPP were determined by monitoring the absorbance change recorded at 410 nm, with 1% DMSO as a control.

### Surface plasmon resonance (SPR) technology based assay

The binding affinity of NPLC0393 to PP2Cα was evaluated by using a Biacore 3000 instrument (Biacore AB, Uppsala, Sweden). Immobilization of the purified PP2Cα to the hydrophilic carboxymethylated dextran matrix of the sensor chip CM5 (Biacore) was performed by the standard primary amine coupling reaction. PP2Cα (8.28 µg/mL in 10 mM sodium acetate, pH 4.2) was then injected over the surface until a desired immobilization level (6000 RU) was reached. Binding affinity measurements were carried out in a continuous flow of 20 µl/min HBS (10 mM HEPES, 150 mM NaCl and 0.005% (v/v) surfactant P20, pH 7.4) as the running buffer. NPLC0393 was diluted in the running buffer and automatically injected in a series of increasing concentrations. The binding responses were recorded continuously in resonance units (RU) at a frequency of 1 Hz as sensorgrams and presented as a function of time. Sensorgrams were processed by using automatic correction for nonspecific bulk refractive index effects. The dissociation equilibrium constant (K_D_) was estimated by the 1∶1 Langmuir binding fit model encoded in the Biacore analysis software.

### Isothermal Titration Calorimetry (ITC) technology based assay

Binding of NPLC0393 to PP2Cα was also measured at 25°C by using a VP-ITC calorimeter (MicroCal, Northampton, MA). All the samples were dialyzed against ITC buffer (50 mM Tris-HCl, pH 7.0 and 1 mM Mn^2+^) and degassed prior to titration. 1.43 ml of 50 µM PP2Cα was titrated by 300 µl of 500 µM PP2Cα using 30 injections. The heat of dilution of NPLC0393 was measured by titrating NPLC0393 into the ITC buffer and was subtracted for data analysis. Data were analyzed with Origin 7.0 software (MicroCal) using a single-site binding model.

### Statistical analysis

All the experiments were repeated at least three times. Data were presented as mean ± SD. Statistical analysis was performed using one-way ANOVA followed by Bonferroni's multiple comparison tests. p value of less than 0.05 was considered statistically significant.
